# NorUDCA promotes degradation of α1-antitrypsin mutant Z protein by inducing autophagy through AMPK/ULK1 pathway

**DOI:** 10.1371/journal.pone.0200897

**Published:** 2018-08-01

**Authors:** Youcai Tang, Keith S. Blomenkamp, Peter Fickert, Michael Trauner, Jeffrey H. Teckman

**Affiliations:** 1 Pediatrics and Biochemistry, Saint Louis University, and Cardinal Glennon Children’s Medical Center, St. Louis, Missouri, United States of America; 2 Pediatrics and Science & Education, the Fifth Affiliated Hospital, Zhengzhou University, Zhengzhou, China; 3 Research Unit for Experimental and Molecular Hepatology, Division of Gastroenterology and Hepatology, Department of Internal Medicine, Medical University of Graz, Graz, Austria; 4 Division of Gastroenterology and Hepatology, Department of Internal Medicine III, Medical University of Vienna, Vienna, Austria; Univerzitet u Beogradu, SERBIA

## Abstract

Alpha-1 Antitrypsin (α1AT) Deficiency is a genetic disease in which accumulation of α1AT mutant Z (α1ATZ) protein in the ER of hepatocytes causes chronic liver injury, liver fibrosis, and hepatocellular carcinoma. No effective medical therapy is currently available for the disease. We previously found that norUDCA improves the α1AT deficiency associated liver disease by promoting autophagic degradation of α1ATZ protein in liver in a mouse model of the disease. The current study unravels the novel underlying cellular mechanism by which norUDCA modulates autophagy. HTOZ cells, modified from HeLa Tet-Off cells by transfection with the resulting pTRE1-ATZ plasmid and expressing mutant Z proteins, were studied in these experiments. The role of norUDCA in inducing autophagy, autophagy-mediated degradation of α1ATZ and the role of AMPK in norUDCA-induced autophagy were examined in the current report. NorUDCA promoted disposal of α1ATZ via autophagy-mediated degradation of α1ATZ in HTOZ cells. Activation of AMPK was required for norUDCA-induced autophagy and α1ATZ degradation. Moreover, mTOR/ULK1 was involved in norUDCA-induced AMPK activation and autophagy in HTOZ cells. Our results provide novel mechanistic insights into the therapeutic action of norUDCA in promoting the clearance of α1ATZ *in vitro* and suggest a novel therapeutic approach for the treatment of α1ATZ deficiency disease and its associated liver diseases.

## Introduction

Alpha-1 Antitrypsin (α1AT) Deficiency is a genetic disease, which is caused by homozygosity for the Z mutant of α1AT and occurs in 1 out 2,000–5,000 live births in North America [1]. The mutant Z protein is encoded by the α1AT mutant Z (α1ATZ) gene, a substitution of lysine for glutamate at residue 342. The protein product adopts a polymerized conformation and aggregates in the ER of hepatocytes (insoluble aggregates), rather than being appropriately secreted into the serum. Accumulation of the α1ATZ protein in liver cells of homozygous individuals triggers an intracellular injury cascade, cell death and chronic liver damage, including fibrosis and hepatocellular carcinoma (HCC). There is no specific pharmacological/medical treatment for homozygous α1ATZ associated liver disease. Therefore, research identifying strategies that reduce accumulations of α1ATZ and/or promote degradation of α1ATZ is of high priority [2]. Hepatocytes cope with the burden of accumulated intracellular protein by activating endoplasmic reticulum-associated proteasomal degradation (ERAD) pathways, which dispose of newly synthesized, soluble, monomeric α1ATZ molecules, and by macroautophagy (called autophagy hereafter), which predominantly targets the large, insoluble accumulations of the aggregated (sometimes called “polymerized”) α1ATZ protein [3–8]. Autophagy is a cellular self-eating process initiated by formation of autophagic vacuoles with double layer membranes to engulf cytoplasmic components (damaged organelles and abnormal protein cargo) for degradation. After formation, the outer membrane of an autophagosome fuses with a lysosome to form an autolysosome, and then the cytoplasmic components are delivered to the lysosomes where the autophagosome-delivered contents and its inner membrane are digested by the lysosome's hydrolases [9]. Autophagy can also determine cell fate and is controlled by autophagy-related genes (ATGs) and protein complexes such as LC3, regulated by various cell signaling molecules, such as phosphatidylinositol 3-kinase (PI3K)/AKT, mammalian target of rapamycin (mTOR) [10] and AMP-activated protein kinase (AMPK) [11]. The kinase mTOR is a critical regulator of autophagy induction, with activation of mTOR (AKT and PI3K signaling) suppressing autophagy, and negative regulation of mTOR (AMPK signaling) promoting it [11, 12]. However, additional experiments still need to elucidate the underlying mechanisms in specific circumstances.

Ursodeoxycholic acid (UDCA) is a minor constituent of human bile [13, 14]. Purified UDCA has various cellular effects described *in vitro*, including anti-apoptotic and anti-inflammatory activity [15, 16]. UDCA is sometimes used off-label in α1AT deficiency patients, although there are no studies confirming its clinical utility. There is also no understanding of the possible therapeutic mechanisms of UDCA in α1AT, except for poorly defined “cell protective” effects [17, 18]. Nor-Ursodeoxycholic Acid (norUDCA) is a side-shortened C23 homologue of UDCA undergoing cholehepatic shunting instead of enterohepatic circulation resulting in profound hepatic enrichment [19, 20]. In various *in vivo* studies, it has been shown to have anti-inflammatory, anti-cholestatic and anti-fibrotic properties greater than UDCA [16, 20, 21, 22, 23]. Our previous data showed potent effects of norUDCA in reversing the liver disease associated with α1AT deficiency in a mouse model *in vivo* [24]. We found that norUDCA had an effect on the intracellular processing and degradation of α1ATZ in an animal model of α1AT liver disease [24]. Our data also showed that the α1ATZ disappearance was associated with increased autophagy by EM quantitation in the PiZ livers [24], although the intracellular mechanisms were not identified. Therefore, the current report is aimed to investigate the underlying molecular mechanisms norUDCA in reducing accumulation and promoting degradation of α1ATZ and to address the underlying mechanisms *in vitro*.

## Materials and methods

### Animals

Euthanasia of mice was performed by carbon dioxide affixation and subsequent cervical dislocation under the expressed approval and guidelines of the IACUC of the Saint Louis University Comparative Medicine Department. This research was approved by the IACUC of the Saint Louis University Comparative Medicine Department. Approval# 1525.

### Chemicals and antibodies

NorUDCA is a gift from Dr. Peter Fickert (Medical University of Graz, Graz, Austria), which was originally received as a gift from Dr. Falk Pharma (Freiburg, Germany). NorUDCA vehicle was water. 5-Aminoimidazole-4-carboxamide ribonucleotide (AICAR), an AMPK activator, was purchased from Sigma (St. Louis, MO). AICAR vehicle was water. 6-[4-(2-Piperidin-1-ylethoxy) phenyl]-3-pyridin-4-ylpyrazolo-[1, 5-a]-pyrimidine (Compound C) was purchased from MCE MedChem Express. Compound C vehicle was DMSO. Chloroquine (CQ) was purchased from Sigma (cat#c-6628). Chloroquine vehicle was water. MG132 was purchased from SelleckChem.com (No.S2619). MG132 vehicle was DMSO. Doxycycline was purchased from Sigma-Aldrich (cat# D3072, USA). Doxycycline vehicle was water.

### Cell culture

HTOZ cells were modified from HeLa Tet-Off cells by transfection with the resulting pTRE1-ATZ plasmid (Teckman et al., 2001). HTOZ/M were cultured in Dulbecco’s modified Eagle’s medium (DMEM) supplemented with 10% of fetal bovine serum (FBS) and Pen/Strep, at 5% CO2, 100% humidity and 37°C. Cells cultured in medium containing doxycycline (DOX, 40ng/ml) turns off expression of α1ATZ proteins. 60–70% confluent cells were used in experiments. In some experiments, Z expression was turned on 24 hours before intervention and sometimes it was simultaneous depending on interaction of drugs and the amount of intracellular protein accumulated required for the readout, for example, depending on if the experiment is to show a prevention or a reduction. When expression is turned on, new mRNA reaches a steady state in approximately 24h, which roughly corresponds to steady state levels of new ATZ monomer protein levels in the ER. However, progressive accumulation of ATZ insoluble aggregates (polymers) continues to increase over several days and is somewhat variable between parent cell lines. The ATZ soluble and insoluble protein fractions have different intracellular half-lives as previously shown [24]. mRNA expression remains stable for 30–40 days then begins to decline as lines age and are passed. Passing cells alters the soluble/insoluble ATZ protein ratios, given the different half-lives of the pools and depending on the age of the cells. Cells were appropriately treated with norUDCA as indicated.

### Western blotting analysis

Whole cell or liver tissue lysates were prepared as previously. Proteins were separated by SDS-PAGE, transblotting and subsequent immunoreactions using chemiluminescence. The following antibodies, including polyclonal rabbit anti-LC3 (1:1000, Cat# NB100-2220, Novus Biologicals), monoclonal rabbit anti-ATG5 (1:500, Cat# ab108327, Abcam, USA), rabbit anti-ATG5-ATG12 complex (1:500, cat# 214526, US Biological), mouse anti-p62 (1:2,500, Cat# 610833, BD Transduction Laboratories), polyclonal goat anti-rabbit IgG-HRP (1:10,000, Cat# p0448, Dako), and donkey anti-mouse IgG-HRP (1:100,000, Cat# sc-2314, Santa Cruz), polyclonal rabbit anti-phospho-AKT (Ser473) (Cell Signaling, #4060), polyclonal rabbit anti-AKT1/2/3 (sc-8312), rabbit anti-phospho-mTOR (Ser2448) (cell signaling, #5536), and rabbit anti-mTOR (cell signaling, #2983), rabbit anti-phospho-AMPK (Thr172) (cell signaling, #2535), and rabbit anti-AMPK (cell signaling, #5832), were used in the current report. Rabbit anti-human α1AT from Immunology Consultants Laboratory (1:1000, cat#RA1T-80-1, 4°C overnight), goat anti- human α1AT from Diasorin (1:25,000, Stillwater, MN, RT 25 minutes), and polyclonal Rabbit anti-goat secondary antibody/HRP was purchased from Dako (Cat#: P0449, 1:50,000, RT 30 minutes). GAPDH was used as an invariant control for equal loading. Densities of bands in Western blotting analyses were normalized with the internal invariable control. Levels of target protein bands were densitometrically determined by using ImageJ 1.47v (Wayne Rasband, National Institutes of Health, USA). Variations in the density were expressed as fold changes compared with the control in the blot.

### RNA extraction and Real-time PCR

Total RNAs were extracted by TRI^®^ Reagent (Sigma, MO, USA) from HTOZ/M cells and treated with DNase I before the synthesis of the first strand of cDNA. Real-time PCR were performed as we previously described using SYBR Green Supermix [25]. mRNA levels are expressed as fold changes after normalization with glyceraldehyde-3-phosphate dehydrogenase (GAPDH), as described by Schmittgen et al. [26]. The primers for human are as follows: α1AT, forward, 5’-GGC CAT ACC CAT GTC TAT CC-3’ and reverse, 5’-TTC ACC ACT TTT CCC ATG AA-3’; p62, forward, 5’-GAC TAC GAC TTG TGT AGC GTC-3’ and 5’- AGT GTC CGT GTT TCA CCT TCC-3’; ATG5, forward, 5’- AGA AGC TGT TTC GTC CTG TGG-3’ and reverse, 5’- AGG TGT TTC CAA CAT TGG CTC-3’; and GAPDH, forward, 5’- GGA GCG AGA TCC CTC CAA AAT-3’ and reverse, 5’- GGC TGT TGT CAT ACT TCT CAT GG-3’.

### Plasmids, transient transfection and LC3 puncta analyses

The plasmid pEGFP-LC3 (Addgene plasmid 21073) was purchased from Addgene (Cambridge, MA, USA). pEGFP-LC3 contained a fragment (738 bp) of the gene LC3. The cells were fed with fresh medium or treated with norUDCA at appropriate dosages in fresh medium for 1 hour prior to transfection. For transfection, semi-confluent cells in p60 dishes were transiently transfected with a total of 3 μg DNA per dish, using the Lipofectamine® 2000 reagent (Invitrogen Corp., Carlsbad, CA), and following the protocol provided by the manufacturer. Cells were fixed with 4% paraformaldehyde at 0, 16 and 24 hours after transfection and observed under inverted fluorescent microscope (Leica DMI 4000 B). The 10–12 areas were randomly chosen and images were taken under 20X magnification. Total living, LC3-positive and puncta-positive cells were counted, based on morphological and fluorescent criteria, and then calculated for percentage of LC3 positive and puncta positive cells, respectively. Only fluorescent cells were included for puncta percentage analysis. Of this population, cells having at least three morphologically discrete fluorescent puncta qualified to be counted as puncta positive cells. The number of puncta positive cells was divided by the Total LC3-GFP positive cells for calculating the percentage.

### ATG5 shRNA plasmid transfection and puromycin selection to produce HTOZ ATG5 knock-down (ATG5-KD) cell line

HTOZ cells were seeded in 6-well plates and grew up to 80% confluent for transfection. Lipofectamine® 3000 (cat# 100022049, Invitrogen) was applied to the transfection protocol following the instructions provided by the manufacturer. Briefly, dilute Lipofectamine® 3000 Reagent (3 μl) in Opti-MEM® (125 μl, cat#31985–070, Gibco® by Life Technologies^TM^, USA). Prepare master mix of DNA as following description: by diluting DNA plasmids (Santa Cruz, USA) ATG5 shRNA plasmid (h) (1 μg, cat# sc-41445), control shRNA plasmid-A (1 μg, cat# sc-108060), and copGFP control plasmid (1 μg, cat# sc-108083), respectively, in Opt-MEM (125 μl). Add diluted DNA to each tube of Lipofectamine® 3000 Reagent (1:1 ratio) and incubate for 5 minutes at room temperature. Add DNA-lipid complex to cells and incubate for 2 days. Visualize copGFP control plasmid transfected cells under inverted fluorescent microscope to estimate transfection efficiency. Add puromycin (final concentration, 2μg/ml) to ATG5 shRNA plasmid and control shRNA plasmid-A transfected cells for 24 hours for drug selection. Do western blot to analyze ATG5 knock-down efficacy.

### Statistical analysis

Differences between means were evaluated using an unpaired two sided Student’s t test (P<0.05 considered as significant). Where appropriate, comparisons of multiple treatment conditions with controls were analyzed by ANOVA with the Dunnett’s test for post hoc analysis.

## Results

### Exogenous norUDCA enhances degradation of α1ATZ in HTOZ cells

**Fig 1:**
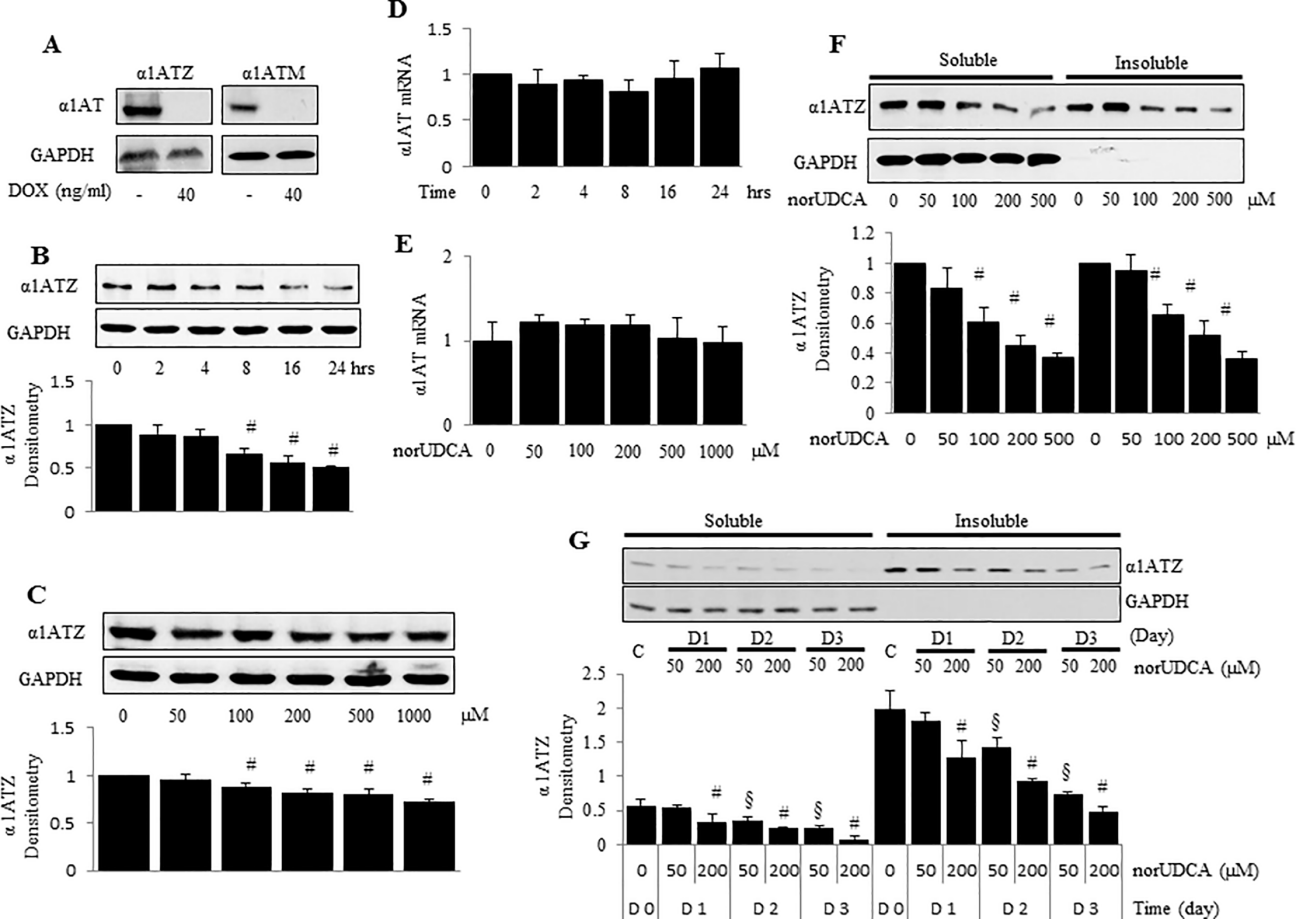
norUDCA reduces the steady-state protein levels, but does not change the mRNA levels of α1ATZ in HTOZ cell line. **A.** Z protein expression in HTOZ or M expression at 24h in HTOM cells is turned off by doxycycline (DOX, final concentration 40ng/ml), which are called HTOZ dox+ or HTOM dox+ cells. **B.** Western blotting analysis of α1ATZ in HTOZ cells after 24h expression at time 0 treated with norUDCA at 200 μM as indicated time points. **C.** Western blotting analysis after 24h expression of α1ATZ in HTOZ cells treated with norUDCA at different doses for 24 hours. **D & E.** Real-time PCR analysis of α1ATZ mRNA levels, 24h expression at time 0, in HTOZ cells treated with norUDCA at 200 μM for indicated time points (D) or at different doses for 24 hours (E). Values are expressed as mean ± SD, n = 3. Representative is from three independent experiments. **F.** Western blotting analysis of insoluble and soluble fractions of α1ATZ at 24h expression in HTOZ cells treated with norUDCA at different doses for 24 hours. **G.** Western blotting analysis of insoluble and soluble fractions of α1ATZ after 72h expression in HTOZ cells passed and seeded at lower density to allow for time course, treated with norUDCA at different doses for 1–3 days. For B, C, F, and G, lower panel showed the densitometric analysis. Values are expressed as mean ± SD after normalization over GAPDH, #<0.05 vs untreated cells, §<0.05 vs d1 (50μM). GAPDH was used as equal loading control. Representative is from three independent experiments.

Our previous data in an in vivo model showed that norUDCA prevented hepatic accumulation of α1AT mutant Z protein polymers in juvenile animals, reversed existing accumulation in adult animals, and reduced liver injury. To test the role of norUDCA in a reduction of accumulation of α1AT mutant Z proteins in an *in vitro* system, HTOZ cells that express mutant Z protein (Fig 1A), were treated with norUDCA at 200 μM for indicated periods (Fig 1B), or at different concentrations (0–1000μM) for 24 hours (Fig 1C) in 10% FBS DOX-free medium. NorUDCA significantly reduced the steady-state levels of α1AT in a time-dependent manner, and to some degree dose-dependently (Fig 1). NorUDCA had no impact on α1AT mRNA levels (Fig 1D and 1E), indicating that norUDCA did not affect α1ATZ synthesis.

Next, we sought to explore whether norUDCA affect the soluble and insoluble fractions of α1ATZ in HTOZ cells. The soluble α1ATZ represents newly synthesized polypeptides, which are known to fold inefficiently during biogenesis and to be retained in the ER. α1ATZ polypeptides in the ER that escape degradation by the ERAD pathway aggregate (polymerize) into insoluble masses targeted for degradation by autophagy. To test differences in the degradation of these two pools by norUDCA, HTOZ cells were treated with norUDCA at 200 μM for 24 hours, soluble and insoluble α1ATZ were isolated for western blotting analysis. This published technique is known to efficiently separate the insoluble aggregates from soluble monomeric α1ATZ molecules [5, 24]. The insoluble fraction is then denatured, which breaks the aggregates (also called polymers) back into monomeric α1ATZ, as they are not bound by covalent bonds. NorUDCA remarkably decreased the steady-state levels of α1ATZ in both the insoluble and soluble fractions *in vitro* (Fig 1F). Similar results were found in the long-term treated HTOZ cells (Fig 1G). Taken together, these data demonstrate that norUDCA reduces accumulation of α1ATZ in HTOZ cells *in vitro*.

### NorUDCA increased autophagy in HTOZ cells

**Fig 2:**
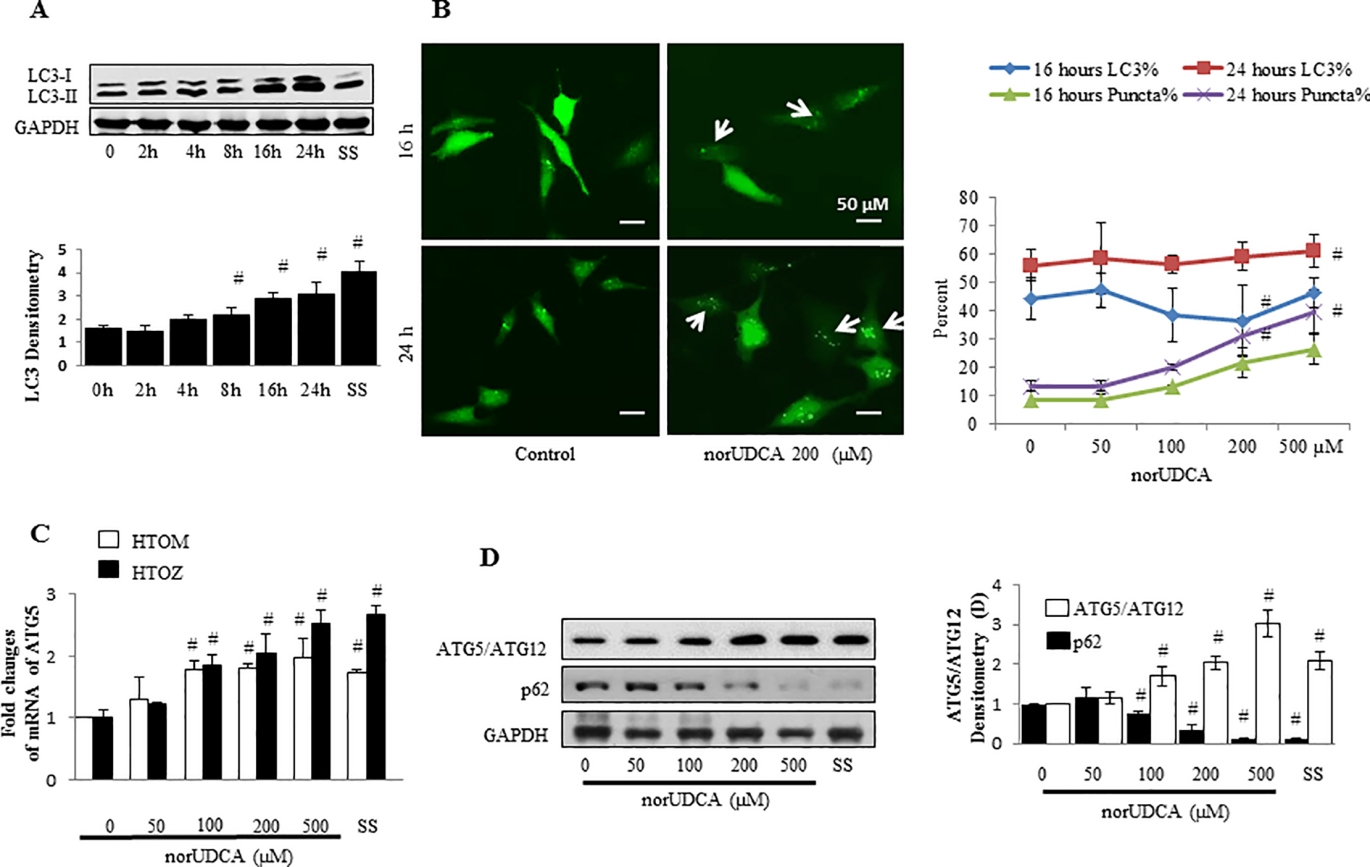
NorUDCA induces autophagy in vitro. **A.** Western blotting analysis of LC3 in HTOZ cell line treated with norUDCA at 200 μM for different time points. **B.** LC3 puncta analysis, the left panel: LC3 puncta was shown in pLC3-EGFP plasmid transfected HTOZ cells followed by treatment with/out norUDCA for 16 hours or 24 hours (original magnification 40X); the right panel: percentage of LC3 puncta positive cells was out of total LC3-EGFP positive cells. Data is expressed as mean ± SD. Representative is from three independent experiments. **C.** Real-time PCR analysis of ATG5 mRNA levels in HTOZ or HTOM cell line treated with norUDCA at different concentrations for 24 hours, data is present as fold changes comparing to untreated cells and expressed as mean ± SD, #<0.05 vs control group, n = 3. SS was used as positive control. Representative is from three independent experiments. **D.** Western blotting analysis of ATG5-ATG12 complex and p62 in HTOZ cell line treated with norUDCA at different concentrations for 24 hours. Representative comes from three independent experiments. For **A** and **D,** the adjacent panels show the respective densitometry analysis. Data is expressed as mean ± SD, #<0.05 vs untreated cells. GAPDH was used as a loading control and normalization. For **A, C** and **D**, SS is used as positive control.

It is well known that misfolded proteins can be degraded through autophagy. To address underlying mechanisms by which norUDCA reduces accumulation of α1ATZ, we test the role of norUDCA in enhancing autophagy, specifically its effect on isoform conversion of autophagosomal membrane-specific protein LC3, an indicator of autophagosome formation, in the HTOZ cell line. Cells were treated with norUDCA at different concentrations or 200μM for different time points as indicated in doxycycline-free DMEM. Whole cell lysates were prepared and analyzed with western blotting analysis. The ratio of LC3-II to LC3-I was increased in both time-dependent manner (2A) and dose-dependent manner (2B) in HTOZ cell line (Fig 2). Also, norUDCA enhanced LC3 puncta formation, another indicator of autophagic activity, in a dose-dependent manner in HTOZ cells after transfection of pEGFP-LC3 plasmids (Fig 2B). LC3 is found in the membrane of the autophagic vacuoles and increased labeling is shown to be proportional to increased vacuole formation in various systems.

Next, we tested if norUDCA affects expression of genes related to autophagy. HTOZ or HTOM cells were treated with norUDCA at different concentrations for 24 hours in doxycycline-free DMEM. Autophagy-related gene 5 (ATG5) is necessary for autophagy due to its role in autophagosome elongation and p62 is commonly used as a marker to study autophagic flux. Both are major genes relevant to autophagy. Real-time PCR analysis showed that norUDCA increased ATG5 mRNA levels both in HTOZ and HTOM cells (Fig 2C and 2D) and western blotting analysis showed that role of norUDCA in increasing ATG5 while reducing p62 protein abundance in HTOZ cells (2D). These data indicate the role of norUDCA in inducing autophagy *in vitro*. Collectively, these data demonstrate that norUDCA induces autophagy *in vitro*.

### NorUDCA increases degradation of insoluble α1ATZ Via induction of autophagy

**Fig 3:**
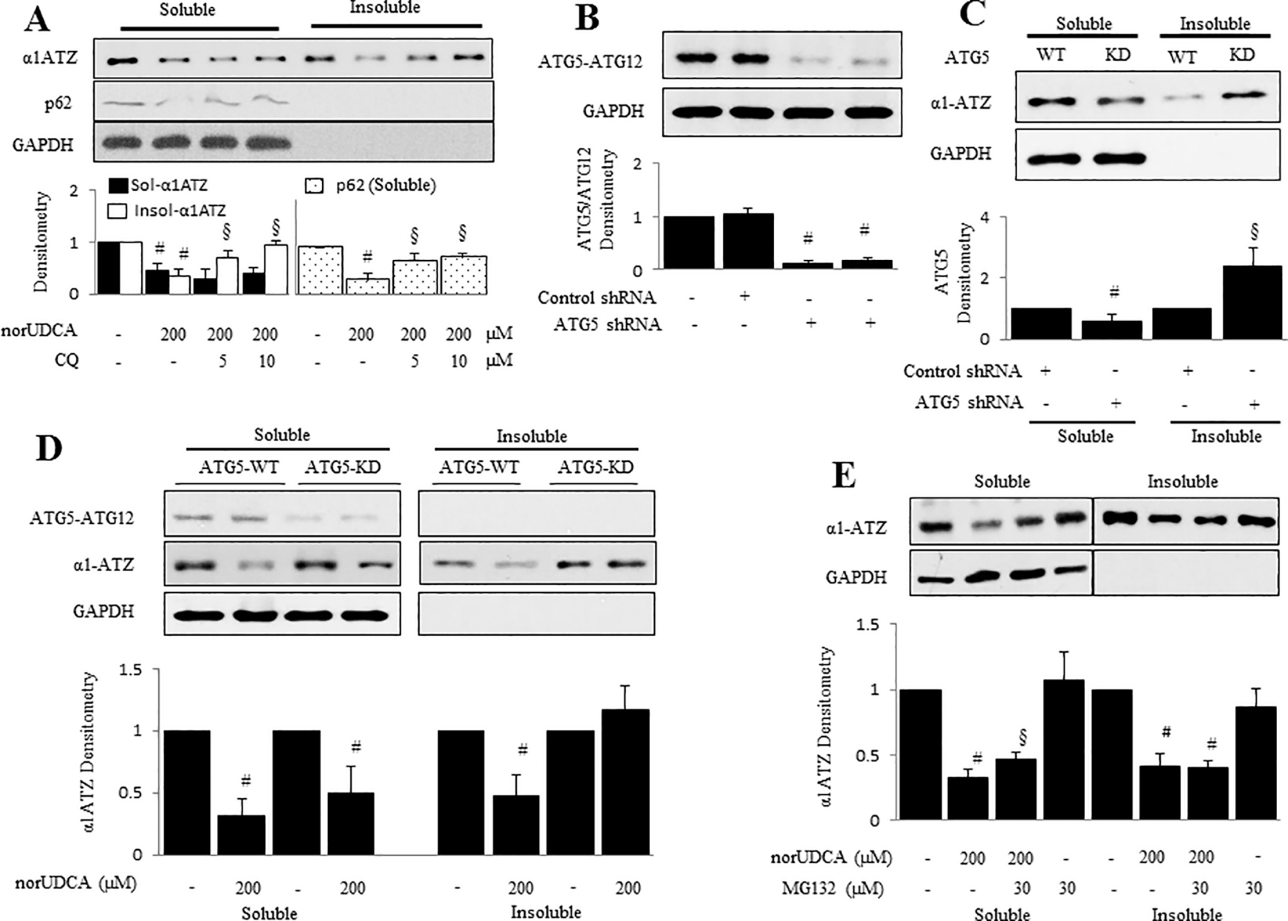
NorUDCA reduces insoluble α1ATZ via inducing autophagy in HTOZ cell line. **A.** Western blotting analysis for soluble and insoluble fraction of α1ATZ in HTOZ cells pretreated with chloroquine (CQ), commonly used pharmacological autophagy inhibitor, at different concentrations (0, 5, 10 μM) for 1 hour before addition of norUDCA at 200 μM for an additional 24 hours. p62 was detected to show that autophagy was blocked by CQ. **B.** Western blotting analyzes the efficacy of ATG5 knock-down. The specific band showed the ATG5-ATG12 complex (56 kD). **C.** Western blotting analysis for soluble and insoluble fraction of α1ATZ in ATG5 expressing (ATG5-WT) or ATG5 knock-down (ATG5-KD) HTOZ cells. **D**. Western blotting analysis for soluble and insoluble fraction of α1ATZ in ATG5-WT or ATG5-KD HTOZ cells treated with/out norUDCA at 200 μM for 24 hours. **E.** Western blotting analysis for soluble and insoluble fraction of α1ATZ in HTOZ cells pretreated with MG132 (30 μM) for 1 hour and subsequently incubated with norUDCA (200 μM) for an additional 24 hours. The cells that were incubated with MG132 alone served as control to validate that the proteasome was inhibited. For A, B, C, D and E, the lower panels show the densitometry of specific bands normalized with GAPDH. Data are expressed as mean ± SD, #<0.05 vs control or ATG5 expressing cells (C), §<0.05 vs norUDCA treated cells. Representatives come from three independent experiments. GAPDH was used as an equal loading control.

To determine if norUDCA-induced autophagy is involved in insoluble α1ATZ degradation, HTOZ cells, cultured in 10% FBS dox-free DMEM up to 80% confluence, were pretreated with chloroquine (CQ), commonly used pharmacological autophagy inhibitor, at different concentrations (0, 5, 10 μM) for 1 hour before addition of norUDCA at 200 μM for an additional 24 hours. Insoluble and soluble α1ATZ was separated for western blotting analysis. As expected in Fig 3A, norUDCA induced a decrease in levels of insoluble α1ATZ in HTOZ cells, which was inhibited by CQ, indicating that norUDCA-induced degradation of α1ATZ was reversed by the blockade of autophagy in vitro.

To confirm effect of norUDCA on α1ATZ degradation involved in enhanced autophagy, we determined its effect on α1ATZ levels in ATG5-KD (atg5 knock-down) HTOZ cells. ATG5^-^KD HTOZ cell line was established by using ATG5 shRNA plasmids to knock down atg5 (autophagy-deficient) as described in Materials and Methods. Importantly, the efficacy of ATG5 knock-down is more than 90% (Fig 3B). Expression of shATG5 blocks autophagy at a proximal step by preventing the formation of the ATG5-ATG12 complex, which is required for the generation of autophagosomes. Knock-down of ATG5 resulted in an increase of insoluble fraction of α1ATZ in ATG5-KD HTOZ cells, compared to ATG5 expressing (ATG5-WT) HTOZ cells (Fig 3C). Further, ATG5 HTOZ and ATG5-KD HTOZ cells were treated with/out norUDCA at 200 μM for 24 hours, respectively. NorUDCA induced a decrease in levels of insoluble α1ATZ in the ATG5-WT HTOZ cells but not in the ATG5-KD HTOZ cells, shown in Fig 3D. NorUDCA also induced a decrease in levels of soluble α1ATZ in both ATG5-WT and ATG5-KD cells. Collectively, the data indicate that norUDCA enhances removal of insoluble α1ATZ by autophagy and has an independent effect on the removal of soluble α1ATZ by mechanism(s) that do not involve the conventional autophagic pathway. Moreover, these data suggest that norUDCA-induced removal of insoluble α1ATZ could be eliminated by blockage of autophagy.

Next, we explored whether ERAD is related to norUDCA-caused reduction of insoluble α1ATZ in HTOZ cell line. The cells, cultured in 10% FBS DOX minus DMEM up to 80% confluence, were pre-incubated with MG132 (30 μM) for 1 hour and followed by treatment of norUDCA (200 μM) for additional 24 hours. Insoluble and soluble α1ATZ was separated for western blotting analysis. Cells that were incubated with MG132 alone served as control to validate that the proteasome was inhibited. As shown in Fig 3E, norUDCA decreased levels of insoluble α1ATZ, which could not be restored by blockage of proteasomal pathway, indicating that norUDCA-mediated decrease in levels of insoluble α1ATZ is independent of proteasomal pathway. Taken together, norUDCA induces degradation of insoluble α1ATZ via autophagic pathway in HTOZ cells. Our previous work shows soluble α1ATZ to be degraded by ERAD^8^, which we did not further investigate here.

### Activation of AMPK mediates norUDCA-induced autophagy and α1ATZ degradation

**Fig 4:**
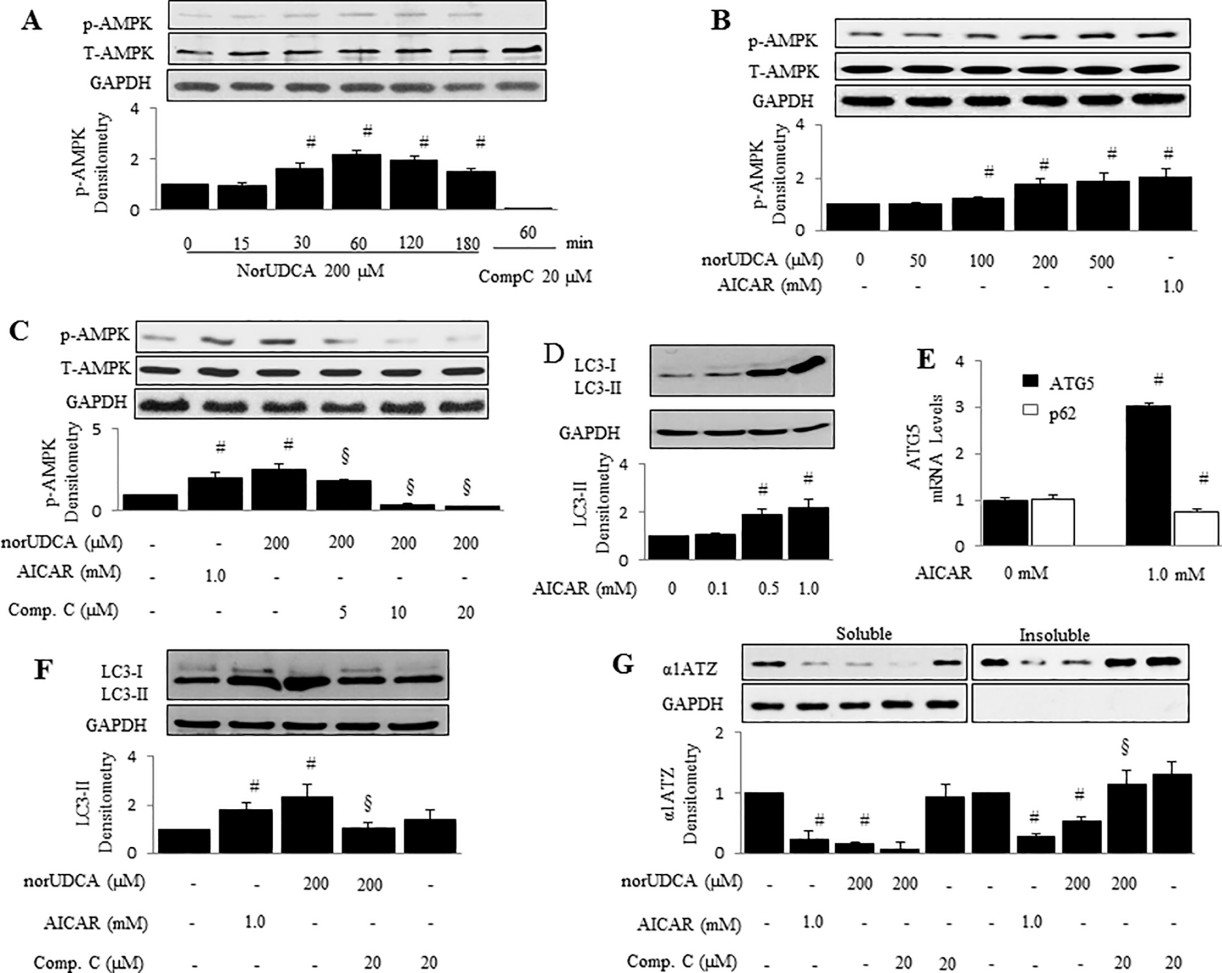
AMPK mediates norUDCA-induced α1ATZ polymer reduction and autophagy in HTOZ cells. **A & B**. phosphorylation of AMPK (Thr172) in HTOZ cells treated with norUDCA at 200 μM for indicated time points (A) or at different doses for 1 hour (B) was determined by western blotting analyses. Compound C (20 μM) or AICAR (1.0 mM) was used as negative or positive control. Representative is from three independent experiments. **C**. Phosphorylation of AMPK (Thr172) in HTOZ cells pretreated with compound C at different concentrations (0–20 μM) for 1 hour prior to the presence of norUDCA at 200 μM for additional 1 hour was determined by western blotting analyses. AICAR (1.0 mM) was used as positive control. For A, B and C, the lower panels are the densitometry of phospho-AMPK after normalization with total AMPK. GAPDH is used as a loading control. Data is expressed as mean ± SD, #<0.05 vs untreated cells, and §<0.05 vs norUDCA treated cells alone. Representative is from three independent experiments. **D**. LC3 in HTOZ cells treated with AICAR at different concentrations (0–1.0 mM) for 24 hours was determined by western blotting analyses. **E**. mRNA levels of ATG5 and p62 were determined by real-time PCR in HTOZ cells treated without/with AICAR at 1.0 mM for 24 hours. Data is expressed as mean ± SD, # < 0.05 vs control, n = 3. **F**. LC3 in HTOZ cells treated with AICAR at 1.0 mM or norUDCA at 200 μM with/out compound C at 20 μM for 24 hours was determined by western blotting analyses. For D and F, the lower panels are the ratio of LC3-II/LC3-I. Data is expressed as mean ± SD, #<0.05 vs untreated cells, and §<0.05 vs norUDCA treated cells alone. Representative is from three independent experiments. **G**. A1ATZ polymers and monomers were isolated and determined by western blotting analyses in HTOZ cells treated with AICAR at 1.0 mM or norUDCA at 200 μM with/out compound C at 20 μM for 24 hours. The lower panel is the densitometry of α1ATZ after normalization with GAPDH. Data is expressed as mean ± SD, #<0.05 vs untreated cells, and §<0.05 vs norUDCA treated cells alone. GAPDH is used as an equal loading control. Representative is from three independent experiments.

AMPK is a sensor of energy in mammalian cells. It has been reported that AMPK regulates autophagy in several cell types [27–28]. Therefore, we sought to examine whether AMPK mediates norUDCA-caused autophagy and α1ATZ polymer reduction, and whether the activator of AMPK, AICAR would initiate this effect. To test this hypothesis, HTOZ cells were treated with norUDCA at 200 μM as indicated time points or at different concentrations (0–500 μM) for 1 hour. Phosphorylation of AMPK (Thr172) was determined by western blotting. As shown, norUDCA increased levels of phosphorylation of AMPK in both the time-dependent manner (Fig 4A) and dose-dependent manner (Fig 4B). NorUDCA-induced AMPK could be dose-dependently eliminated by Compound C, a well-established pharmacological inhibitor of AMPK (Fig 4C), showing that norUDCA can activate AMPK *in vitro*.

Next, we tested whether AICAR, mimics the effect of norUDCA and induces autophagy in HTOZ cells. The cells were treated with AICAR at different concentrations (0–1.0 mM) for 24 hours. As shown in Fig 4D, AICAR increased LC3-II protein abundance. Moreover, data from quantitative real-time PCR indicated that AICAR increased mRNA levels of ATG5 while slightly decreased p62 mRNA levels, the major autophagy related genes, in HTOZ cells after exposure to AICAR at 1.0 mM for 24 hours (Fig 4E). To establish the role of AMPK in mediating norUDCA-induced autophagy, HTOZ cells were pretreated with/without compound C (20 μM) for 1 hour prior to addition of norUDCA (200 μM) or AICAR (1.0 mM) for 24 hours. Western blotting analyses indicated that norUDCA increased the ratio LC3-II/GAPDH and LC3-II/LC3-I, which was abrogated by compound C (Fig 4F) *in vitro*. These data demonstrate that activation of AMPK mediates norUDCA-induced autophagy in HTOZ cells.

Further, we tested the role of AMPK in mediating norUDCA-induced α1ATZ polymer reduction in HTOZ cells. The cells were pretreated with/without compound C (20 μM) for 1 hour prior to addition of norUDCA (200 μM) or AICAR (1.0 mM) for 24 hours. Isolation of α1ATZ polymer and monomer was performed for western blotting analyses. As shown in Fig 4G, norUDCA, mimic AICAR (the 2nd and 7th lane from the left), reduced α1ATZ polymers and monomers (the 3rd and 8th lane from the left), which was repressed by compound C (the 4th and 9th lane from the left). There was no change between control (The 1st lane) and compound C alone (the last lane). These results suggest that AMPK signaling is involved in norUDCA-induced α-ATZ reduction. Taken together, our results demonstrate that activation of AMPK mediates norUDCA-induced autophagy and α1ATZ degradation in HTOZ cells *in vitro*.

### Involvement of mTOR signal in norUDCA-induced AMPK activation and autophagy

**Fig 5:**
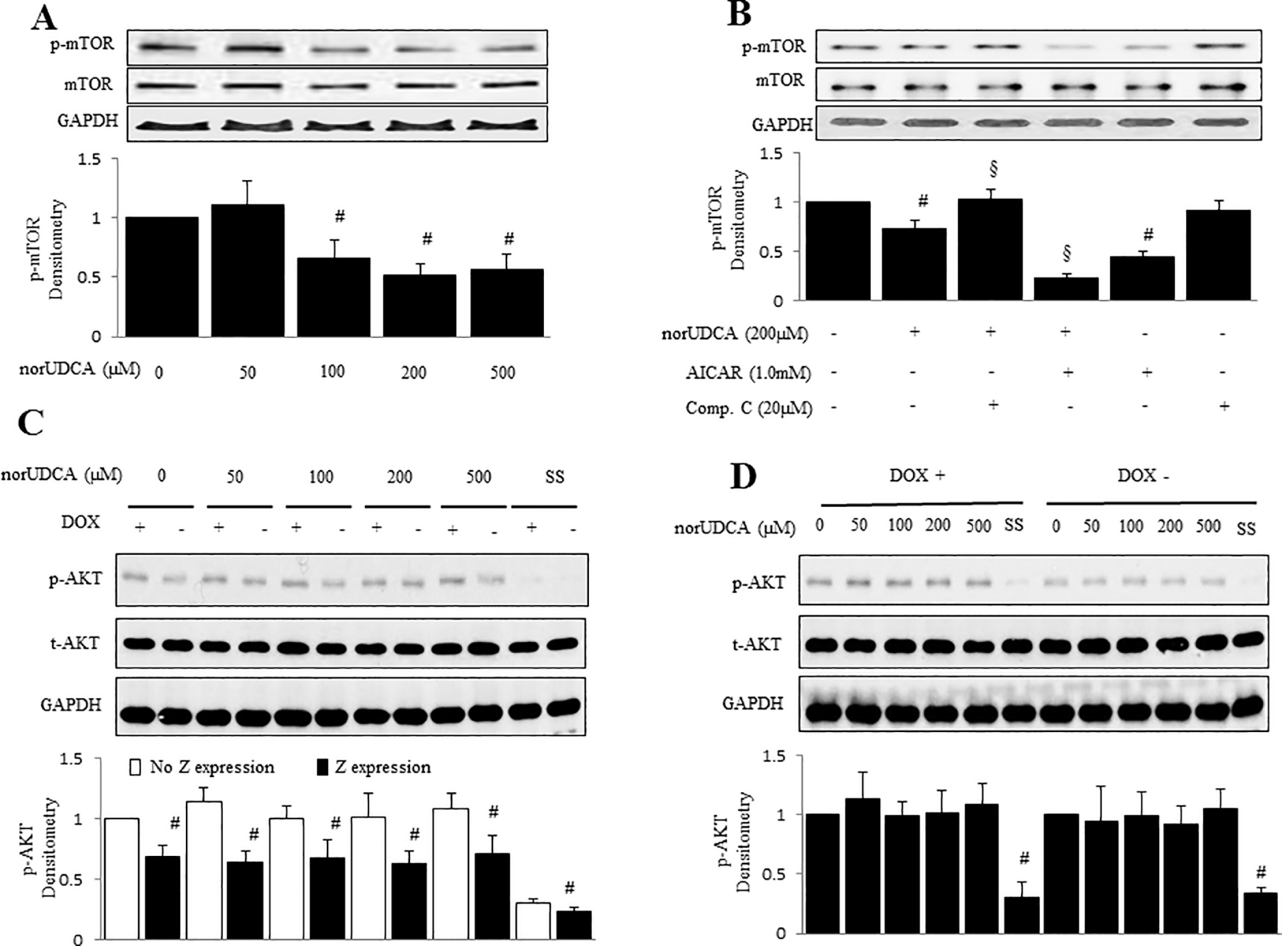
NorUDCA modulates mTOR via AMPK in HTOZ cells. **A**. Western blotting analysis for phosphorylation of mTOR (Ser2448) in HTOZ cells after treatment with norUDCA for 1 hour. **B**. Western blotting analysis for phosphorylation of mTOR (Ser2448) in HTOZ cells after pretreatment with AICAR or compound C for 1 hour and then in the presence or absence of norUDCA for an additional 1 hour. For A and B, the lower panels are the densitometry of phospho-mTOR (Ser2448) after normalization with total mTOR. Data is expressed as mean ±SD, #<0.05 vs untreated cells, §<0.05 vs norUDCA treated cells. Total mTOR is used for normalization and GAPDH is used as a loading control. Representative is from three independent experiments. **C & D**. Western blotting analysis for phosphorylation of AKT in HTOZ dox+ or HTOZ dox- cells after treatment with norUDCA for 1 hour. The lower panels are the densitometry of phosphorylation of AKT after normalization with total AKT. Data is expressed as mean ± SD, #<0.05 vs no Z expression (C) or untreated cells (D). GAPDH is used as a loading control. SS is used as negative control.

To further address mechanisms by which norUDCA enhances degradation of α1ATZ proteins via AMPK activation in HTOZ cells, we evaluated the downstream of AMPK signaling related to autophagy, such as mTOR, a serine/threonine protein kinase that involves in autophagy, and the signals upstream of mTOR, including AKT. The HTOZ cells were treated with norUDCA at different concentrations for 1 hour. As shown by western blotting analysis, norUDCA decreased phosphorylation of mTOR Ser2448 (Fig 5A), indicating that mTOR might be involved in norUDCA-induced AMPK activation. To establish the role of mTOR in norUDCA-mediated AMPK activation, HTOZ cells were pretreated with AICAR or compound C for 1 hour and subsequently treated with or without norUDCA at 200 μM for an additional 1 hour. As shown in western blotting analysis, norUDCA decreased phosphorylation levels of mTOR Ser2448 (Fig 5B), which was suppressed by compound C and synergistically enhanced by AICAR, suggesting that norUDCA modulates mTOR via AMPK signaling pathway. However, norUDCA did not alter the levels of AKT phosphorylation (Fig 5C and 5D). Collectively, our data demonstrates that mTOR is involved in norUDCA-induced AMPK activation.

### Activation of ULK1 is needed For norUDCA-induced reduction of insoluble fraction of α1ATZ Via AMPK activation

**Fig 6:**
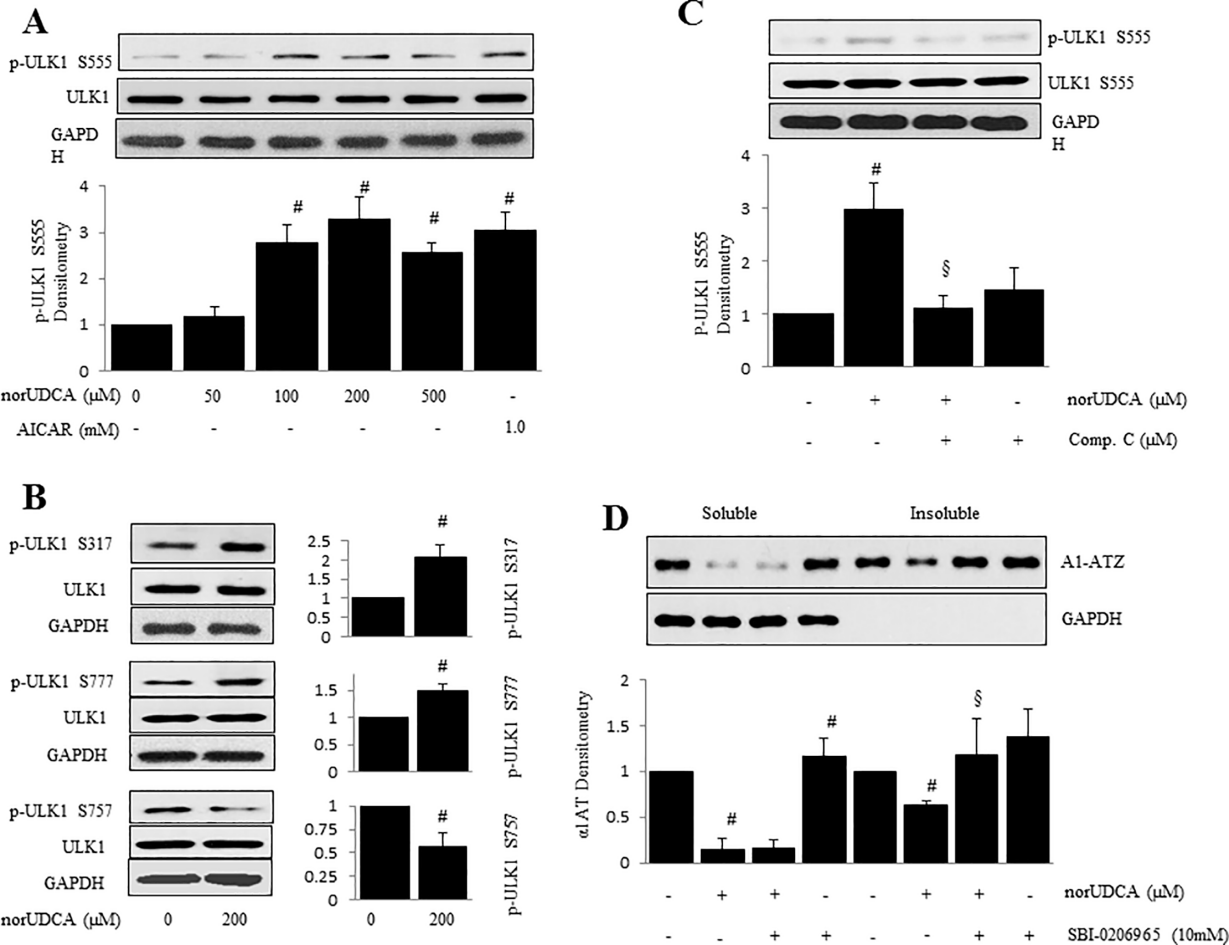
Activation of ULK1 is required for norUDCA-induced AMPK activation and reduction of polymers of α1ATZ. **A.** Western blotting analysis for phosphorylation of ULK1 (Ser555) in HTOZ cells treated with norUDCA at different concentrations for 1 hour. The lower panel is the densitometry of phospho-ULK1 Ser555 after normalization with total ULK1. Data is expressed as mean ± SD, #<0.05 vs untreated cells. AICAR is used as positive control. **B.** Western blotting analysis for phosphorylation of ULK1 Ser317, Ser757 and Ser777 in HTOZ cells treated with norUDCA at 200 μM for 1 hour. The left panels are the densitometry of p-ULK1 after normalization with total ULK1, #<0.05 vs untreated cells. **C**. Western blotting analysis for phosphorylation of ULK1 S555 in HTOZ cells pretreated with compound C (10 μM) for 1 hour and followed by absence or presence of norUDCA at 200 μM for an additional 1 hour. The lower panel is the densitometry of p-ULK1 Ser555 after normalization with total ULK1. Data is expressed as mean ± SD, #<0.05 vs untreated cells and §<0.05 vs norUDCA alone treated cells. Compound C is used as negative control. For A, B and C, total ULK1 is used for normalization and GAPDH is used as a loading control. **D**. Polymers and monomers of α1ATZ were determined by western blotting analysis in HTOZ cells pretreated with SBI-0206965 (10mM), inhibitor of ULK1, for 1 hour prior to addition of norUDCA at 200 μM for additional 24 hours. The lower panel is the densitometry of α1ATZ after normalization with GAPDH. Data is expressed as mean ± SD, #<0.05 vs untreated cells, §<0.05 vs norUDCA alone treated cells. GAPDH is used as loading control and for normalization.

Unc-51 like autophagy activating kinase 1 (ULK1) is an important protein in autophagy and plays a critical role in early steps of autophagosome biogenesis. AMPK upregulates ULK1 activity via AMPK-dependent phosphorylation [11, 29] and hence is essential in induction of autophagy [30]. To further address mechanisms by which norUDCA enhances autophagy and degradation of α1ATZ proteins in HTOZ cells, we sought to evaluate whether norUDCA activates ULK1 via AMPK signaling pathway, and whether ULK1 mediates norUDCA-induced reduction of insoluble fraction of α1ATZ in HTOZ cells. The HTOZ cells were treated with norUDCA at different concentrations for 1 hour. As shown by western blotting analysis, norUDCA increased phosphorylation of ULK1 at Ser555 site (Fig 6A), also increased phosphorylation of ULK1 at Ser317, Ser777 sites and decreased Ser757 (Fig 6B), indicating that norUDCA activates ULK1, which might be involved in norUDCA-induced autophagy.

To establish the role of ULK1 in norUDCA-mediated AMPK activation, HTOZ cells were pretreated with AICAR or compound C for 1 hour and subsequently treated with or without norUDCA at 200 μM for an additional 1 hour. As shown in western blotting analysis, norUDCA increased phosphorylation of ULK1 Ser555, which was suppressed by compound C (Fig 6C), suggesting that norUDCA induces ULK1 via activation of AMPK signaling pathway.

To further establish the role of ULK1 in norUDCA-mediated clearance of insoluble fraction of α1ATZ, HTOZ cells were pretreated with SBI-0206965 (10 mM), ubiquitous inhibitor of ULK1, for 1 hour and followed by treatment with or without norUDCA at 200 μM for an additional 24 hours. Insoluble and soluble α1ATZ was separated for western blotting analysis. As shown in western blotting analysis, norUDCA decreased insoluble fraction of α1ATZ, which was diminished by SBI-0206965 (Fig 6D), suggesting that norUDCA-induced ULK1 is involved in the removal of insoluble α1ATZ. Collectively, our data demonstrates that norUDCA induces autophagy and reduces accumulation of α1ATZ by modulating AMPK/ULK1 signaling pathway.

## Discussion

In homozygous ZZ individuals, the classic form of α1AT deficiency, mutant Z protein synthesized in the liver misfolds, aggregates and accumulates in hepatocyte ER, leading to cell dysfunction, susceptibility to cell death, and liver injury [2, 31]. Liver transplantation is the only curative approach for patients with severe α1AT deficiency disease. In the current work, we demonstrate that norUDCA reduced accumulation of α1ATZ and promoted degradation of aggregates (polymers) of α1ATZ by inducing autophagy via activation of AMPK/mTOR/ULK1 signaling pathway.

NorUDCA is a derivate of UDCA that is a minor constituent of human bile. Both norUDCA and UDCA indicate the therapeutic potential against cholestatic and metabolic disorders [15, 19–21, 32]. Since norUDCA is only effectively conjugated with glycine or taurine, it has specific physicochemical and therapeutic properties distinct from UDCA [21]. Our previous work showed that norUDCA ameliorated α1AT deficiency associated liver disease in a mouse model. Here, HTOZ cell line was applied to the experiments as previously described [8]. While not a liver cell line, several past publications have established this cell line as useful and have shown that it faithfully recapitulates the intracellular processing and degradation of mutant Z protein. In fact, every aspect of Z protein intracellular retention, trafficking and degradation that has been possible to test in cell culture, mouse liver and human liver have shown the same results. Here we found that norUDCA lowered the steady-state and polymers of α1ATZ, but did not alter synthesis of α1AT in HTOZ cell line (Fig 1), which is indicative that norUDCA effectively reduced accumulation of α1ATZ by promoting degradation in an in vitro system. This is the first study showing the role of norUDCA on the intracellular mechanisms of α1ATZ degradation. These data confirm the hypothesis that norUDCA enhanced degradation of α1ATZ, leading to disposal of the burden of α1ATZ, and appeal to us to further address the underlying mechanisms.

Wild type α1AT (“M” allele) is predominantly synthesized in liver and rapidly secreted into the blood to protect host tissues from the enzyme neutrophil elastase during periods of inflammation. The Z mutation causes the freshly produced α1AT polypeptide chain to be misfolded and retained within ER of hepatocytes, resulting in hepatic damage. For mammalian cells, misfolded Z protein can be targeted by autophagy and ERAD [5–8]. We previously reported that the proteasome plays an important role in ER degradation of α1ATZ in many cell types including hepatocytes [8]. Our laboratory and others have shown the responsibility of autophagy for efficient disposal of ER-retained α1ATZ [5]. Therefore, targeting autophagy and proteasome pathways could be an effective approach to removal of α1ATZ. As an explanation for norUDCA-induced degradation of α1ATZ in HTOZ cells (Fig 1), we found that norUDCA induced autophagy in HTOZ cells (Fig 3), which is required for norUDCA-mediated degradation of polymers of α1ATZ other than proteasome pathway (Fig 4). NorUDCA also decreased levels of soluble α1ATZ in both wild-type and atg5-deficient HTOZ cells, suggesting that norUDCA enhances removal of insoluble α1ATZ by autophagy and has an independent action on the removal of soluble α1ATZ by a mechanism that is not involved in the autophagic pathway.

Autophagy is important for balancing sources of energy at critical times in development and in response to nutrient stress [33]. Usually, autophagy is activated under cellular stress and nutrient deprivation and is regulated by cell signaling pathways, including PI3K/AKT, mTOR and AMPK [10–12]. Our data showed that norUDCA-activated AMPK was required for norUDCA-induced autophagy and reduced polymers of α1ATZ (Fig 5). AMPK is a Serine/Threonine kinase that plays a role in cellular energy homeostasis and activated by a rise in the ratio of AMP/ATP within the cell upon depletion of ATP [34]. In addition, AMPK kinase activity is upregulated by LKB1 [35, 36] and Ca2+/calmodulin-dependent protein kinase kinase-β (CaMKKβ) [37, 38]. Although the exact mechanism by which norUDCA activates AMPK is not clear, it can be explained by the possibility of norUDCA in increase of Ca^2+^ [39] in the cells. Additional experiments are needed to address this issue.

**Fig 7:**
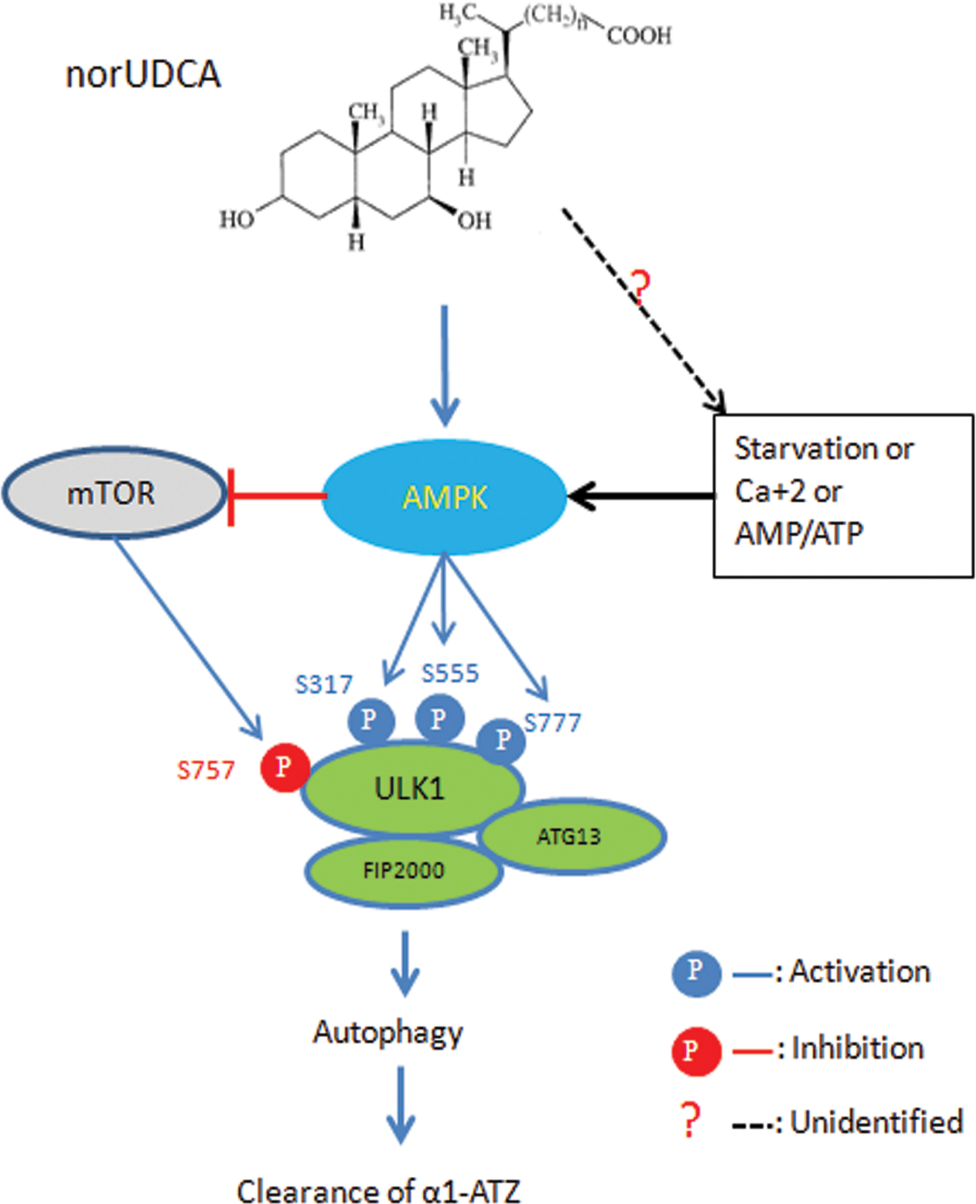
Schema of proposed mechanisms by which norUDCA promotes degradation of α1ATZ. NorUDCA activates AMPK and inhibits mTOR. AMPK phosphorylates ULK1 at Ser317, Ser555 and Ser777, and inhibits Ser757, and subsequently initiates autophagy, resulting in degradation of polymers of α1ATZ and reduction of accumulation of α1ATZ in hepatocytes.

This study also unraveled that norUDCA increased phosphorylation of ULK1 at Serine (Ser) 317, Ser555 and Ser777 sites, and suppressed phosphorylation of mTOR, which was reversed by blockade of AMPK signaling pathway, but norUDCA did not affect phosphorylation of AKT (Fig 6), showing the involvement of mTOR and ULK1 in this process. ULK1 is the initiator of autophagy, which has multiple phosphorylation sites, including Ser317 & Ser777 [40], Ser555 [41, 42] and Ser757 [43]. The former three are AMPK-dependent and the last is associated with mTOR. The direct interaction between AMPK and ULK1 positively regulates ULK1 activity through AMPK-dependent phosphorylation [11] and is essential for autophagy induction [30]. Bach et al. confirmed Ser555 as a major AMPK-dependent phosphorylation [41]. Based on the current observations, we propose that the mechanism by which norUDCA promotes degradation of polymers of α1ATZ is to induce autophagy by activating AMPK, and subsequently by phosphorylating and activating ULK1 and inhibiting mTOR signaling pathway, leading to the reduction of accumulation of α1ATZ in hepatocytes in vitro (Fig 7). Future work will examine if norUDCA affects ERAD and other degradation pathways of soluble α1ATZ.

Increasing evidence shows that autophagy can exert as a promising target to treat a number of pathological conditions, such as neurodegeneration [44] and aging [45]. Increased mTOR activity is associated with development of Alzheimer’s disease (AD) in vitro and in vivo models [46], and with Huntington disease in animals [47] and humans [48]. We previously reported that rapamycin, an inhibitor of mTOR and activator of autophagy, showed its ability to reduce α1ATZ protein accumulation and ameliorate other markers of liver injury in PiZ mice [49]. However, not all the α1AT aggregates in the cell were fully degraded, for reasons that are still not clear, and an unusual dosing scheme was required to see benefits in the mouse model. Given that these mechanistic and toxicity questions remain, human studies of rapamycin have not commenced [50]. In contrast to other drugs previously proposed for α1AT deficiency [7, 49], norUDCA has the main advantage of being safe and well tolerated and is currently undergoing further clinical development in humans [16, 51]. Our findings could be rapidly translated into a clinical trial. In conclusion, our results in this report shed novel insights into mechanisms of norUDCA in the reduction of accumulation of α1ATZ in vitro and provide a therapeutic candidate for the treatment of α1AT deficiency disease and its associated liver diseases.
